# *N*-Butyl-cyanoacrylate-assisted retrograde transvenous obliteration (NARTO) for gastric varices

**DOI:** 10.1016/j.radcr.2024.04.058

**Published:** 2024-05-18

**Authors:** Eisuke Shibata, Hidemasa Takao, Yusuke Watanabe, Osamu Abe

**Affiliations:** Department of Radiology, The University of Tokyo, Graduate School of Medicine, 7-3-1 Hongo, Bunkyo-ku, Tokyo 113-8655, Japan

**Keywords:** Balloon-occluded retrograde transvenous obliteration, Gastric varix, Gastrorenal shunt, *n*-butyl cyanoacrylate

## Abstract

We describe the usefulness of *n*-butyl-cyanoacrylate (nBCA)-assisted retrograde transvenous obliteration (NARTO) for gastric varices in 3 consecutive patients. In all patients, balloon catheters were inserted into the gastrorenal shunt via the left renal vein. After injecting sclerosant into the gastric varix under balloon occlusion, nBCA was injected to the proximal side of the shunt, to completely embolize the shunt. NARTO is a simple technique to achieve stagnation of the injected sclerosant in gastric varices and to occlude a gastrorenal shunt. This procedure is also cost-effective, and may improve procedure time compared with original or modified balloon-occluded retrograde transvenous obliteration.

## Introduction

Gastric varices (GVs) are serious complications of portal hypertension that are found in about 20% of patients with liver cirrhosis [1]. Although GVs are less frequent than esophageal varices, bleeding from gastric varices is associated with higher morbidity and mortality rates than bleeding from esophageal varices [2]. Several treatments have been developed for GVs including endoscopic, endovascular treatment, and surgical treatment. Although endoscopic and endovascular treatment are less stressful to the patients than surgery, the choice of endoscopic or endovascular treatment depends on the complex nature of GVs, the patient's condition, and locally available resources. Endoscopic treatment is limited by potential adverse events such as endoscope damage, systemic embolization or infection. As endovascular procedures for GVs, balloon-occluded retrograde transvenous obliteration (BRTO) and transjugular intrahepatic portosystemic shunt (TIPS) are recommended as first-line therapies for secondary gastric variceal bleeding prophylaxis, although BRTO may cause increase in portal pressure, and TIPS is associated with postprocedural hepatic encephalopathy [1].

Since BRTO was originally reported by Kanazawa et al, it has become a well-established treatment for GVs and is less invasive than surgery and is associated with a lower recurrence rate than endoscopy sclerotherapy [3]. However, for conventional BRTO, the balloon must be kept for 4–24 hours after sclerosant injection to prevent sclerosant leakage. Therefore, several modifications of BRTO have been reported to overcome the length of the procedure and the burden to patients. Gown et al. reported plug-assisted retrograde transvenous obliteration, which shortened the procedure time by using a vascular plug, although it was associated with a relatively high rate of GV recurrence [4]. Lee et al. reported coil-assisted retrograde transvenous obliteration (CARTO), which used coils instead of a vascular plug or balloon catheter to shorten the procedure [5]. Furthermore, Kim et al. introduced CARTO-II [6], in which coils are placed under balloon occlusion just after sclerosant injection to the GVs.

We have experienced a case of BRTO in which the gastrorenal shunt (GRS) was not successfully embolized using a small number of coils after sclerosant injection. To lower the cost and shorten the procedure time, we injected *n*-butyl cyanoacrylate (nBCA) to embolize the GRS after sclerosant injection (case 1). Before now, the use of nBCA in BRTO was previously reported in a case report, in which it was injected after injecting a foam sclerosant into a GV via a brachial vein approach [7]. In this report, we describe three consecutive patients in whom GVs were treated by nBCA-assisted retrograde transvenous obliteration (NARTO), after a case (case 1) in whom nBCA was injected after coil placement.

## Case reports

### Case 1

**Fig. 1:**
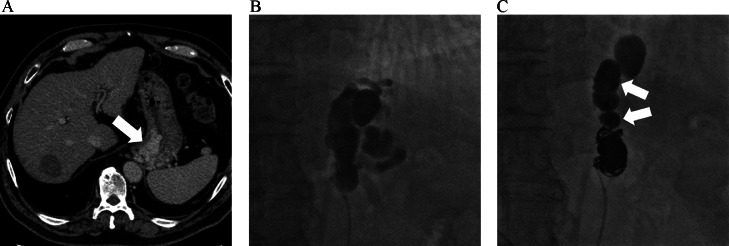
A 72-year-old patient with gastric varices (case 1). (A) Contrast enhanced computed tomography before *n*-butyl cyanoacrylate-assisted retrograde transvenous obliteration showed gastric varices protruding into the stomach (arrow). (B) After inserting a balloon catheter into the gastrorenal shunt, 5% ethanolamine oleate iopamidol was injected under balloon occlusion. (C) Because the insertion of 11 long coils did not completely embolize the gastrorenal shunt, *n*-butyl cyanoacrylate (arrow) was injected through a microcatheter under balloon occlusion.

In a 72-year-old patient with alcoholic liver cirrhosis, GV was detected incidentally by endoscopic screening. Contrast-enhanced computed tomography (CECT) showed a protruding GV with a dilated GRS acting as a drainage vein (Fig. 1A). Endovascular treatment was planned after consultation with gastroenterologists.

An 8 Fr hook-shaped sheath (Supersheath, Medikit, Tokyo, Japan) was inserted into the right femoral vein under local anesthesia with the patient in the supine position. A 5.2 Fr balloon catheter (balloon size, 9 mm; Selecon MP balloon catheter, Terumo Clinical Supply, Tokyo, Japan) was inserted into the GRS. The balloon catheter was retrogradely advanced enough to occlude the GRS. After confirming the blood flow was completely blocked in the GRS and depicting the GV with balloon-occluded retrograde transvenous venography (BRTV), 5% ethanolamine oleate iopamidol (EOI; Oldamin, ASKA Pharmaceutical, Osaka, Japan) mixed with the same volume of nonionic contrast medium (iopamidol 300 mg I/mL, Iopamidol 300, Bayer Schering Pharma, Osaka, Japan) was injected under balloon occlusion using an intravenous drip infusion of 4000 U of haptoglobin (Fig. 1B). Fifteen minutes later, the GRS was embolized using coils (Interlock IDC fibered coil, Boston Scientific, MA, USA; 6 coils of 10 × 50 cm, 2 coils of 8 × 20 cm, 3 coils of 6 × 20 cm) through a 2.5 Fr microcatheter (Renegado, Boston Scientific, MA, USA), but occlusion was not achieved.

To reduce the cost and procedure time, we planned to embolize the GRS using nBCA. After advancing the 2.5Fr microcatheter through the coils, nBCA (nBCA:Lipiodol = 1:4) was injected under balloon occlusion following a small amount of 5% glucose (Fig. 1C). After 2 minutes, the GRS was completely embolized. The patient was discharged without any complications. Two months after treatment, CECT depicted no enhancement of the GVs.

### Case 2

**Fig. 2:**
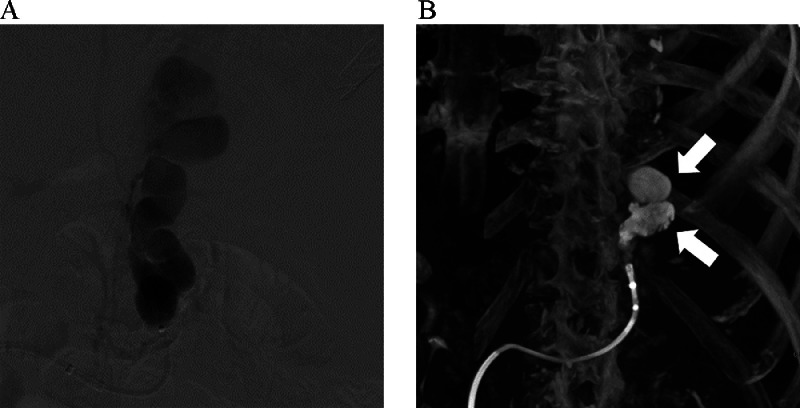
A 71-year-old patient with gastric varices (case 2). (A) After inserting a balloon catheter into the gastrorenal shunt, 5% ethanolamine oleate iopamidol was injected under balloon occlusion. (B) Three-dimensional image derived from cone-beam computed tomography without contrast media showed that injected *n*-butyl cyanoacrylate (arrow) formed a cluster in the gastrorenal shunt under flow control.

A GV associated with hepatic C virus-related liver cirrhosis was found in a 71-year-old male during detailed examination for a suspected gallbladder carcinoma. After consultation with gastroenterologists, endovascular treatment of the GV was planned before surgery for a suspected gallbladder carcinoma. CECT revealed a GV with a dilated GRS as a drainage vein. A 10 Fr hook-shaped sheath (Supersheath) was inserted into the right femoral vein under local anesthesia with the patient in the supine position. A 9 Fr/5 Fr double-coaxial balloon catheter (balloon sizes, 20 and 10 mm; CANDIS, Medikit, Tokyo, Japan) was inserted into the GRS. The smaller balloon catheter was retrogradely advanced enough to occlude the GRS and to depict the GVs. The larger balloon catheter was also located in the GRS at a level sufficient to block blood flow (Fig. 2A). Then, EOI was injected under double-balloon occlusion using 4000 U of haptoglobin. Because of the large diameter of the GRS and insertion of a large number of coils was necessary, we planned to perform embolization using nBCA alone in consideration of its cost-effectiveness and shorter procedure time. A 2.1 Fr microcatheter (Sniper 2 selective, Terumo Clinical Supply) was advanced and nBCA (nBCA:Lipiodol = 1:2) was injected under balloon occlusion following 5% glucose infusion (Fig. 2B). After 2 minutes, complete embolization of the GRS was achieved. Although the patient had slight fever on the next day, he was given conservative antipyretic treatment and discharged 5 days after treatment. Endoscopy performed 2 months after treatment confirmed the GV had shrunk.

### Case 3

**Fig. 3:**
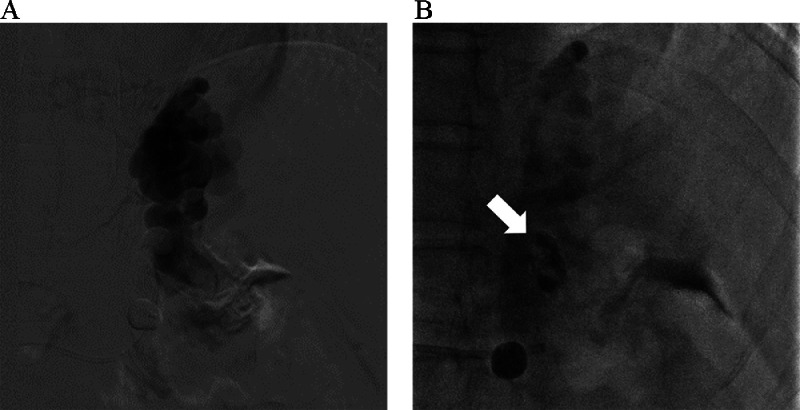
A 60-year-old patient with gastric varices (case 3) (A) After inserting a balloon catheter into the gastrorenal shunt, 5% ethanolamine oleate iopamidol was injected under balloon occlusion. (B) For embolization of the gastrorenal shunt, *n*-butyl cyanoacrylate (arrow) was injected into the gastrorenal shunt through a microcatheter under balloon occlusion, following infusion of 5% glucose.

A GV was found in a 60-year-old male with hepatic C virus-related hepatitis. After consultation with gastroenterologists, endovascular treatment was planned for the GV with a GRS as a drainage vein. A 10 Fr hook-shaped sheath was inserted into the right femoral vein under local anesthesia with the patient in the supine position. A 9 Fr/5 Fr double-coaxial balloon catheter (balloon sizes, 20mm and 10mm; Medikit, Tokyo, Japan) was inserted into the GRS. Although BRTV depicted the GV, it was difficult to advance the balloon catheters enough to spare small collateral drainage veins. Therefore, EOI was injected in a stepwise manner to occlude the small collateral veins first and finally the GV under balloon occlusion with the larger balloon catheter. However, a collateral drainage vein to the GRS distal to the large balloon catheter appeared during EOI injection. Therefore, we stopped injecting EOI. After adjusting the large balloon catheter to block the collateral drainage vein, EOI was again injected into the GV (Fig. 3A).

Because of the large diameter of the GRS, we planned to embolize it with nBCA alone, like in case 2. Therefore, a 2.7 Fr microcatheter (Sniper 2 high flow, Terumo Clinical Supply, Tokyo, Japan) was advanced to the GRS, and nBCA (nBCA:Lipiodol = 1:2) was injected under balloon occlusion following 5% glucose (Fig. 3B). After 2 minutes, complete embolization of the GRS was achieved. The patient was discharged without any complications. One month after treatment, CECT showed no enhancement of the GV.

## Discussion

**Fig. 4:**
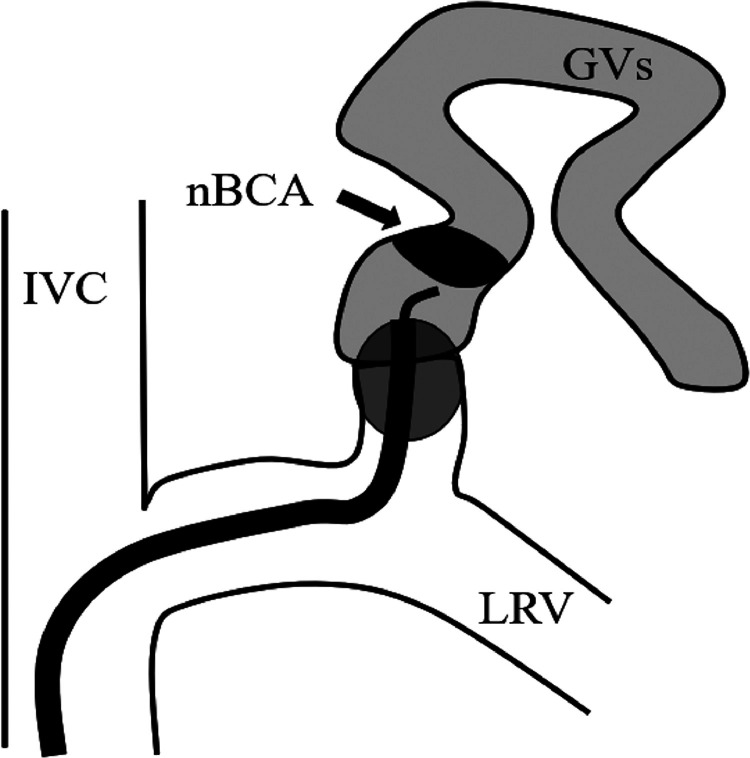
The scheme of *n*-butyl cyanoacrylate-assisted retrograde transvenous obliteration. *n*-butyl cyanoacrylate is injected through a microcatheter under balloon occlusion after injecting the sclerosant into the gastric varices. GV, gastric varix; IVC, inferior vena cava; LRV, left renal vein; nBCA, *n*-butyl cyanoacrylate.

This report describes NARTO for GVs (Fig. 4). NARTO is a cost-effective, time-saving technique compared with the original BRTO or CARTO-II for GVs and represents a “kind” technique for patients and interventionalists. Overnight inflation of an occlusion balloon involves additional hospital resources and more stressful for the patients, and there is a risk of complications associated with a balloon and sheath. NARTO will not be influenced by previous endoscopic treatments, similar to the original and modified BRTO procedures.

NARTO is a simple technique that uses nBCA to embolize the GRS after injecting a sclerosant under balloon occlusion, unlike in CARTO-II, which uses coils [6]. NARTO thus offers advantages in terms of cost-effectiveness and saves time compared with CARTO-II because nBCA is cheaper than coils, and it is quicker to inject nBCA than to insert a sufficient number of coils to occlude the GRS [8]. In addition, because NARTO can be performed independently of the diameter of the GRS, when the GRS can be completely blocked using a balloon catheter. In case 1, we first tried to embolize the GRS by inserting 11 long fibered coils, but complete embolization was not achieved. We think this was because of the large diameter of the GRS.

There are 2 key technical aspects that we need to consider during NARTO: (1) injecting nBCA without it sticking to a balloon catheter; and (2) injecting nBCA to form a large cluster. During nBCA injection, careful positioning of the microcatheter's tip is important to prevent it sticking to the balloon catheter. We injected nBCA through a microcatheter under balloon occlusion following 5% glucose injection. By using a microcatheter, it is possible to maintain a safety margin between the injected nBCA and the balloon catheter. Under balloon occlusion, nBCA first spreads to the side of the GV because nBCA flows to the region with lower pressure. During injection, nBCA hardens on the side of the GV and flows to the side of the balloon. At this point, we slowly pull back the microcatheter and finally stop injecting nBCA. After waiting for 2 minutes, we deflate the balloon catheter.

The second important technical aspect is to make nBCA form a large cluster. We consider that the occlusive effect of nBCA is weaker if it forms small particles rather than a cluster. In addition, if the injected nBCA particles are too small, they may migrate after the balloon is deflated. To allow nBCA to form a cluster, we injected nBCA in a continuous manner. As illustrated in the images for cases 1 and 2, the injected nBCA formed clusters. However, in case 3, the injected nBCA formed a sponge-like appearance, even though we injected nBCA continuously. We suspect that the reason for the sponge-like appearance is that the injected nBCA hardened together with a coagulated thrombus created by the sclerosant.

NARTO is contraindicated if balloon occlusion of the GRS is not possible. Because the sclerosant is injected under balloon occlusion to block the flow of the GRS, nBCA must be injected under similar flow control. Otherwise, the injected nBCA would be pushed by the blood flow to the systemic circulation. nBCA-related complications are potential disadvantages of NARTO when compared with CARTO-II. To prevent nBCA adhering to a balloon catheter, careful positioning of the microcatheter tip during nBCA injection is important. If the injected nBCA spreads to the side of the GV, we can continue injecting sufficient nBCA to occlude the GRS. However, if the injected nBCA flows back to the microcatheter, the microcatheter must be pulled back to the side of the balloon. When nBCA has stuck to the microcatheter, the microcatheter should be pulled back into the balloon catheter to scrape off the nBCA stuck to the microcatheter and to avoid fixation of the microcatheter inside the nBCA cluster. If nBCA has stuck to the balloon catheter, nBCA must be scrape off with the sheath, by pushing the sheath up to the position of the balloon catheter. Another nBCA-related complication is migration of nBCA after balloon deflation. Although this situation is similar to the risk of coil migration in CARTO-II, we consider that injecting a sufficient amount of nBCA is important, and that the interventionalist should wait several minutes to confirm occlusion [9]. After waiting for several minutes, we slowly deflate the balloon to confirm that the injected nBCA has hardened and is fixed at the position. If nBCA migrates, immediate re-occlusion of the balloon and additional embolization are necessary to avoid complications related to nBCA migration such as pulmonary embolism.

There are several limitations of NARTO for GVs. First, NARTO is impossible if the GRS is not completely blocked with a balloon catheter. Second, the number of treated patients is small and accumulated experience of NARTO in more patients is needed to evaluate the safety and efficacy. Third, direct comparisons with other techniques, such as CARTO, and CARTO-II, are needed to establish the usefulness of NARTO in terms of its cost-effectiveness and procedure time.

## Patient consent

Our institutional review board approved the publication of this report. We obtained informed consent from all the patients.
